# Gamete Size Is Essential for Understanding Sex and Sexual Selection in Humans, Animals, Plants, and Algae But Configurations of Sex-Linked Traits Are Practically Important for Understanding Sex

**DOI:** 10.1007/s10508-025-03216-0

**Published:** 2025-08-12

**Authors:** David A. Frederick

**Affiliations:** 1 Crean College of Health and Behavioral Sciences, Chapman University, Orange, CA USA; 2 Department of Psychology, One University Drive, Orange, CA 92866 USA

## It’s All About Gametes: Definition of Sex in Executive Order 14168

On July 17, 2002, a plague ripped across the planet, killing every living animal in the world carrying a Y chromosome. Sometimes referred to as a terrible “gendercide,” “manslaughter,” or “malepocalypse,” all embryos and sperm carrying a Y chromosome died off as well. Only people without a Y chromosome survived, with two exceptions: a young man named Yorick Brown and his male pet capuchin monkey Ampersand. So begins the graphic novel *Y: The Last Man* (Vaughn, 2002).

The novel makes the assumption that the reader will intuitively agree that Y chromosomes are the defining feature of what makes someone a “man” or “male.” In contrast, sex is defined based on gamete size in the recent Executive Order 14,168: *Defending Women from Gender Ideology Extremism and Restoring Biological Truth to the Federal Government* (White House, 2025). The executive order defines a female as “a person belonging, at conception, to the sex that produces the large reproductive cell” and a male as a person who produces the “small reproductive cell.” The order states that sex is an immutable biological trait and distinct from gender identity. It also states that gender identity does not provide a reasonable basis for identification and emerges from a “gender ideology” that permits the “false claim” that males can identify as, and become, women. It declares that the terms male/female should be treated as equivalent to man/woman in interpretation and application of federal law and administration policy.

This prioritization of gamete size in the definition of sex was surprising to many people because it is not how the typical lay person, doctor, or sex researcher thinks about sex. Many other commenters will undoubtedly expertly highlight the legal, social, and moral issues with the erasure of intersex and transgender individuals from the order. I am therefore highlighting the scientific background underlying the focus on gametes in the definition of sex because I suspect this aspect will be covered less extensively by other commentaries.

First, I briefly highlight recent discussions of sex and how they contrast with a gametic central understanding of sex in the executive order. I then provide some background on why gametes have been central to defining sex across species in evolutionary biological sciences. Understanding this perspective is important because it helps understand the competing frames people are using when they talk of sex as binary instead of as a spectrum or a multidimensional configuration of traits. The gametic central framing is essential for understanding sex and sexual selection across species, whereas the multidimensional configuration framing is practically useful in medical, psychological, and social sciences when studying emergent sex-related structures and how they relate to social roles (e.g., Lee, 2018).

## Competing Popular Narratives: What Is a Woman and What Is a Man?

The idea in *Y: The Last Man* that chromosomes determine sex clearly resonated with many people during the Summer 2024 Olympics. Boxer Imane Khelif competed in these games, but during the competition the International Boxing Association claimed she had “failed gender eligibility tests in the past two years” (Sullivan & Al-Kassab, 2024).

The implication was that she had a Y chromosome, which led to a rush of social media commentary about transgender athletes participating in sports. Some commenters presumed she had advantages because of her genes and testosterone levels. Candidate Donald Trump weighed in at rallies, stating “I’d like to congratulate the young woman who transitioned from a man into a boxer. You saw he won. She won the gold medal” (Bezants, 2024). Trump vowed that he would “keep men out of women’s sports” and “it’s so demeaning to women” (Associated Press, 2024). It appeared, however, that many people had mistakenly jumped to the conclusion that Imane was transgender. She responded to these claims by declaring she was a woman from birth and reporters interviewed her father who showed photos of her as a little girl.

The introduction of this new information quickly shifted online discussions to people confidently expressing that Imane was “really” a man because XY chromosomes are what make you male and therefore she was a man. In response, people tried explaining the complexity of intersex conditions (i.e., how a person can be chromosomally XY, have cells in their body that cannot bind to androgens, and therefore appear female-typical and not have advantages in building muscle mass). Still others told both groups to knock it off and stop speculating about her genes and genitals, it was none of their business: how she identifies was all that matters. After winning the gold, Khelif declared “I was born a woman. I lived [as] a woman. I competed as a woman. There’s no doubt about that” and that all of the criticisms had made her win even more special (Sullivan & Al-Kassab, 2024).

Although these competing frames surged in popularity that summer, many other definitions of sex have been used throughout history. The classic “is it a boy or a girl” question was typically answered based on primary sexual characteristics: was the baby born with a penis or a vulva? And then swaddled in a blue or pink blanket to advertise their sex to others.

But when meeting a new person, we (typically) do not know the state of their genitalia, and we make judgments of their sex based on configurations of facial features and secondary sexual characteristics associated with testosterone and estrogen. We also make judgements based on tertiary sex-linked characteristics, such as long or short hair style, wearing a suit or dress, and other cultural markers. Finally, we rely on their self-identification.

On March 28, a reporter asked President Trump to define what a woman is, and his answer was “A woman is someone who can have a baby under certain circumstances. A woman is a person who is much smarter than a man, I’ve always found” (Williams, 2025). This definition focused on ability to give birth offered in the press conference was interesting, however, because it differed from the one provided via executive order.

## Why Is It All About Gametes (Putting Aside the Political Reasons)? Evolutionary Biology and the Role of Gametes in Sexual Selection

Gamete size is used as the starting point for understanding the evolution of sex because it is the only trait that differentiates the sexes across animal, plant, and algae species (Goymann et al., 2023; Griffiths & Spencer, 2025; Lehtonen & Parker, 2014). Taken to the extreme, gametes are sex, and all other traits are correlates of sex. Biology textbooks convey the unique role of gametes with language such as:

“*Sex: It’s About the Gametes*: Attempting to find a distinguishing characteristic that can be universally applied across sexually reproducing organisms is challenging! But, biologically speaking, the distinction is quite simple—sexes can be distinguished, across the board, by *gamete* (or sex cell) size. Sexually reproducing anisogamous species generally have only two sexes. So how do we distinguish the males from the females? Scientists have created a definition of *female* that includes all the individuals that produce large gametes (eggs), those that produce small gametes (sperm) are *male*. Biologically, this large gamete/small gamete distinction between males and females is the only one that holds up well across many sexually reproducing species” (Section 8.4; Cotner & Wassenberg, 2020).

This gamete-centered definition is a helpful starting point because, in anisogamous species, there are only two gamete types: female sex and male sex. The form seen in humans, oogamy, features small motile gametes (sperm) versus large, immotile gametes (egg), is a common arrangement in many species. Some interesting combinations can occur such as “bisexual plants” emerge as that possess both small and large gametes (i.e., two sexes in one plant as a reproductive strategy), but in these species, the only way to create offspring is through the combination of these two gametes.

## Gamete Size: The Driving Force Behind Sex and Sexual Selection

The binary nature of gametic sex is a critically important concept in evolutionary biology. The evolution of different gamete sizes is important for understanding the emergence of what people generally think of as male and female sexes and the accompanying sex-related traits (Goymann et al., 2023; Griffiths & Spencer, 2025).

The splitting of gametes into small, cheap, plentiful, inexpensive, motile gametes versus large and expensive gametes happened across evolutionary time. Undifferentiated gamete cells carried by organisms specialized into two forms: the larger form (egg) that contains most of the cell organelles and resources needed to develop a zygote, whereas the smaller form (sperm) that carries minimal genetic information and resources. Organisms that specialized in egg creation were labeled females, those that specialized in sperm creation were labeled males.

Parental investment theory provides one lens for understanding how sexual selection processes connected to gamete size led to sex differences in phenotypes and behavior (Trivers, 1972). The theory proposes that organisms will be choosier about who they mate with when they invest more in offspring survival and reproduction, because they bear more costs of reproduction. Trivers noted that biological sex is one factor that influences parental investment.

Trivers noted that once there was an initial split in organismal investment in sex cells, there would have been a reinforcing process where larger (female) cells gained advantages over other female cells by continuing to build in greater sources of energy and support for the development of the zygote. Male sex cells would gain advantages over other male sex cells by decreasing their size and cost. As a result, across evolutionary time, “the male’s initial parental investment, that is, his investment at the moment of fertilization, is much smaller than the female’s” (Trivers, 1972, p. 72). Extensive theoretical work has highlighted how competition among gametes led to smaller and smaller male gametes versus large female gametes, to the point where an individual female gamete is now 10 million times larger than an individual male gamete in volume (Johnson et al., 2021).

This relative difference in gamete expense leads to an initial difference in parental investment (costs) between the sexes, which are then the driving force in the development of other sex-related traits, which deliver those gametes, aid in intrasexual competition, or lead to being chosen as a mate. As Goymann et al. (2023) put it, from this gametic central view: “sex is binary, even though there is a rainbow of sex roles” (p. 1) that emerge from this initial sex difference.

## The Narrow Practical Attempt to Fix the Gamete-Central Definition: Including Intersex

This gamete-central definition in the executive order, however, has several technical limitations when applied to humans in everyday life (beyond the obvious downplaying of the critical importance of people’s gender identities). It makes no provision for what happens to people who have mutations or surgeries that prevent creation of gametes. Second, the definition provides no guidance on how you classify a person who is intersex according to this gametic definition (i.e., with a “Disorder of Sexual Development” or “Difference of Sexual Development”). A literal reading of the order would also invalidate the sex assigned at birth framework, since many intersex conditions are not detected at birth.

Many configurations of sex-associated traits can emerge in which someone is male-typical in some respects, but female-typical in others. For example, a person with Swyer syndrome (46,XY pure gonadal dysgenesis) would typically have XY chromosomes and produce no gametes, and would develop internal female-typical internal genitalia and external genitalia (e.g., vulva). By chromosomal sex they would be male, but by the classic “is it a boy or girl” test at birth they would be female, but by the literal text of the executive order they would have no sex.

Multiple states have now passed bills that reinforce the definition provided in this order. Although some of these bills replicate the same technical limitations as the original order, the West Virginia Legislature (2025) made adjustments to partially address them:

“A ‘male’ [‘female’], when this term is used in reference to a natural person, is an individual who naturally has, had, will have through the course of normal development, or would have but for a developmental anomaly, genetic anomaly, or accident, the reproductive system that at some point produces, transports, and utilizes sperm [‘ova’] for fertilization. (7) ‘Sex’, when this term is used to classify or describe a natural person, means the state of being either male or female as observed or clinically verified at birth. There are only two sexes, and every individual is either male or female: *Provided*, That individuals with congenital and medically verifiable “DSD conditions” (sometimes referred to as “differences in sex development,” “disorders in sex development,” or “intersex conditions”) are not members of a third sex and must be accommodated consistent with state and federal law.”

The West Virginia bill, however, then goes on to equate man/woman with male/female, and to reserve certain spaces only for males and females (e.g., restrooms, changing rooms, and sleeping quarters in domestic violence shelters).

## Sex as a Configuration of Traits in Multidimensional Space

An alternative view of sex is that it is a context-dependent multidimensional variable (Miyagi et al., 2021). Whereas gamete size ties back to the ultimate evolutionary origin of sex, this view is more practically useful for fields working with the proximate biological systems in the body. For example, an urologist and obstetrician will likely be focused more on gonads, external genitalia, and internal reproductive system. A geneticist will be more focused on chromosome and specific genes.

The way I introduce the concept to my Human Sexuality class is intended to communicate both the gametic central definition of sex and the multidimensional view point of sex-related traits. I ask the class to consider how and why the following traits are sometimes not all in the male or female-typical direction given individual: their chromosomal sex (XX or XY), genetic sex (SRY genes present or not), gonadal sex (ovaries or testes), internal reproductive system (Wolffian ducts or Mullerian ducts), external genitalia (penis-scrotum or vulva), hormonal sex (testosterone and estrogen levels), brain sex (male-typical or female-typical differentiation), and gametic sex (sperm or egg). Layered on top of these physical manifestations of sex are their psychological experiences of gender and the body. Then I describe why gametes are central from an evolutionary perspective, and why we focus on sex practically and historically as a configuration of traits.

The objection from the gamete central frame is that this “sex practical” or multidimensional configuration frame is that many of these traits are only valid for a given species or group of species (Griffiths, 2021). The sex practical frame also confuses “sex with the outgrowths of sexual differentiation or the developmental processes that lead to the expression of the biological sex” (Goymann et al., 2023, p. 4). After evaluating these and other essays by evolutionary biologists, my reading of the objection being expressed is that this definition should not be abandoned simply because it is currently being appropriated by people who wield the definition in a manner that is harmful. There will always be people who twist a scientific concept to achieve their own political and social ends, and will simply ignore scientific evidence that does not fit with their views (see: COVID pandemic).

At the individual level, however, for practical, legal, and social purposes there is a social decision to be made about what we decide to label “sex” for understanding evolutionary processes versus what we label “sex” or “sex-associated” traits for social, legal, and practical purposes. Some evolutionary biologists have staked out a position that sex belongs to gametes, whereas an alternative perspective is sex belongs to configurations and a different term belongs to gametes.

The gametic centric definition of sex has some practical limitations for studying some topics important to sexuality researchers, such as sexual orientation. Previously, Frederick et al. (2023) proposed that: “attraction to people who produce sperm versus eggs would not map well onto our general understanding of what constitutes a person’s sexual orientation. Furthermore, attractions to many of the different variations of body types within the category of *intersex* and *transgender* are not easily grouped within the often-used categories of gay/lesbian, bisexual, or heterosexual” (p. 287).

There are many complex ways of thinking of sexual orientation. To start with just a simple example, one definition of sexual orientation is the sex(es) your genitals are aroused by or the sex(es) you find attractive. One aspect of sex we are interested in this case is the configuration of primary and secondary sexual characteristics that a person finds attractive or arousing, and regardless of their gamete type. For example, we would likely conclude that a man is heterosexual if he is attracted to a female who has had her ovaries removed. We would likely conclude that man who feels attraction to person walking down the street with XY chromosomes but female-typical primary and secondary sexual characteristics is heterosexual.

However, sexual orientation is complex, and involves beliefs about chromosomal status, genitalia, and hormones. For example, imagine a study where a man’s sexual orientation toward a clothed female-presenting person changes if you systematically vary whether she purportedly has XX vs. XY chromosomes, penis vs. vulva, male-typical vs female-typical hairstyle and clothing in that culture, etc.? If those factors play a role in attraction, it suggests that gametic sex is not the relevant determinant of sexual orientation for those aspects of sexual orientation.

## Concluding Thoughts

Moving forward, researchers will need to come to a consensus on terminology. We accept that some words or phrases like “to cleave” can have literally the exact opposite meanings depending on the situation (to join together in some instances, or to split apart in others). My (personal) answer to “what is a woman” and “what is sex” has been to be okay with that kind of ambiguity. The word woman can refer to a biological female, or it can refer to a person with the gender identity and social roles, personality, interests, and/or gender expression historically associated with females in a culture. Female can refer to an organism that produces (or would produce) large gametes, and/or to configurations of traits typically associated with the sex that produces those gametes within a species, with intersex individuals having configurations of sex-associated traits fall outside female-typical configurations. Perhaps we need different terms for gametic central sexes and multidimensional configuration sexes if context clues are not sufficient to determine meaning.

Sex is binary at the gametic level in humans and millions of other species. The binary nature of gametic sex, however, is not logically relevant to whether or not gender identity is useful as a marker of identity. The executive order attaches corollaries to the gametic central of sex that do not logically follow from usefulness of this definition or understanding. The executive order takes the fact that gametic sex is binary and important and then unnecessarily excludes sex-associated traits from the understanding of sex. The definition provided fails to address intersex people and does not incorporate important scientific biological concepts related to sex. Instead, the language of the order raises the concern that it instead serves a political and social function of selectively relying on specific scientific concepts to reinforce a specific gender ideology.

## Data Availability

Data transparency not applicable.

## Code Availability

Software application or custom code not applicable.
